# Type XVIII collagen degradation products in acute lung injury

**DOI:** 10.1186/cc7779

**Published:** 2009-04-09

**Authors:** Gavin D Perkins, Nazim Nathani, Alex G Richter, Daniel Park, Murali Shyamsundar, Ritva Heljasvaara, Taina Pihlajaniemi, Mav Manji, W Tunnicliffe, Danny McAuley, Fang Gao, David R Thickett

**Affiliations:** 1 Lung Injury and Fibrosis Treatment Program (LIFT), Department of Medical Sciences, The Medical School University of Birmingham, Edgbaston, Birmingham, B15 2TT, UK; 2 Collagen Research Unit, Department of Medical Biochemistry and Molecular Biology, University of Oulu, Oulu, FIN-90014, Finland; 3 Department of Intensive Care, University Hospital Birmingham, Edgbaston, Birmingham, B15 2TH, UK; 4 Department of Anaesthesia and Intensive Care, Birmingham Heartlands Hospital, Birmingham, B9 5SS, UK; 5 Respiratory Medicine Research Cluster, Centre for Infection and Immunity, Microbiology Building, The Queen's University of Belfast, Grosvenor Road, Belfast, BT12 6BN, Northern Ireland, UK; 6 Lung Investigation Unit, Queen Elizabeth Hospital, Birmingham, B15 2TH, UK

## Abstract

**Introduction:**

In acute lung injury, repair of the damaged alveolar-capillary barrier is an essential part of recovery. Endostatin is a 20 to 28 kDa proteolytic fragment of the basement membrane collagen XVIII, which has been shown to inhibit angiogenesis via action on endothelial cells. We hypothesised that endostatin may have a role in inhibiting lung repair in patients with lung injury. The aims of the study were to determine if endostatin is elevated in the plasma/bronchoalveolar lavage fluid of patients with acute lung injury and ascertain whether the levels reflect the severity of injury and alveolar inflammation, and to assess if endostatin changes occur early after the injurious lung stimuli of one lung ventilation and lipopolysaccharide (LPS) challenge.

**Methods:**

Endostatin was measured by ELISA and western blotting.

**Results:**

Endostatin is elevated within the plasma and bronchoalveolar lavage fluid of patients with acute lung injury. Lavage endostatin reflected the degree of alveolar neutrophilia and the extent of the loss of protein selectivity of the alveolar-capillary barrier. Plasma levels of endostatin correlated with the severity of physiological derangement. Western blotting confirmed elevated type XVIII collagen precursor levels in the plasma and lavage and multiple endostatin-like fragments in the lavage of patients. One lung ventilation and LPS challenge rapidly induce increases in lung endostatin levels.

**Conclusions:**

Endostatin may adversely affect both alveolar barrier endothelial and epithelial cells, so its presence within both the circulation and the lung may have a pathophysiological role in acute lung injury that warrants further evaluation.

## Introduction

Acute lung injury (ALI) is characterised by neutrophilic inflammation of the alveolar-capillary barrier. ALI has multiple aetiologies, but appears to follow a uniform pattern of injury at a cellular level. Extensive damage to the alveolar-capillary barrier leads to the influx of a protein-rich oedema fluid and accompanying inflammatory cells into the alveoli. A complex cascade of both inflammatory and anti-inflammatory cytokines is triggered and inflammatory cells, including neutrophils and monocytes, are recruited to the alveoli. Studying these processes early in the course of the disease can be challenging because most insults causing lung injury are not predictably timed.

Two human models of lung injury allow assessment of the early phases of ALI. One lung ventilation (OLV) during oesophagectomy is associated with a significant post-operative risk of ALI with proposed causative mechanisms including the ischaemic/reperfusion insult experienced by the collapsed lung, oxidative stress injury and barotrauma to the ventilated lung [1]. Systemic levels of inflammatory cytokines have been shown to relate to the duration of OLV and to be reduced by temporary ventilation of the collapsed lung [2]. In addition, recently there have been several studies looking at lipopolysaccharide (LPS) challenge in human volunteers. This model induces a significant neutrophilia in the bronchoalveolar lavage (BAL) with disruption of the alveolar-capillary barrier and allows early pathophysiological changes within defined time limits to be assessed.

The neutrophilic inflammation of the alveolar capillary barrier in ALI and the models of OLV or LPS challenge result in the release of proteases including collagenases [3]. The balance between collagen formation and degradation is a complex and dynamic process within the lung of patients with ALI [4]. BAL studies suggest changes in collagen production and degradation may promote collagen deposition within the lung, even at the onset of lung injury [4,5].

**Figure 1:**
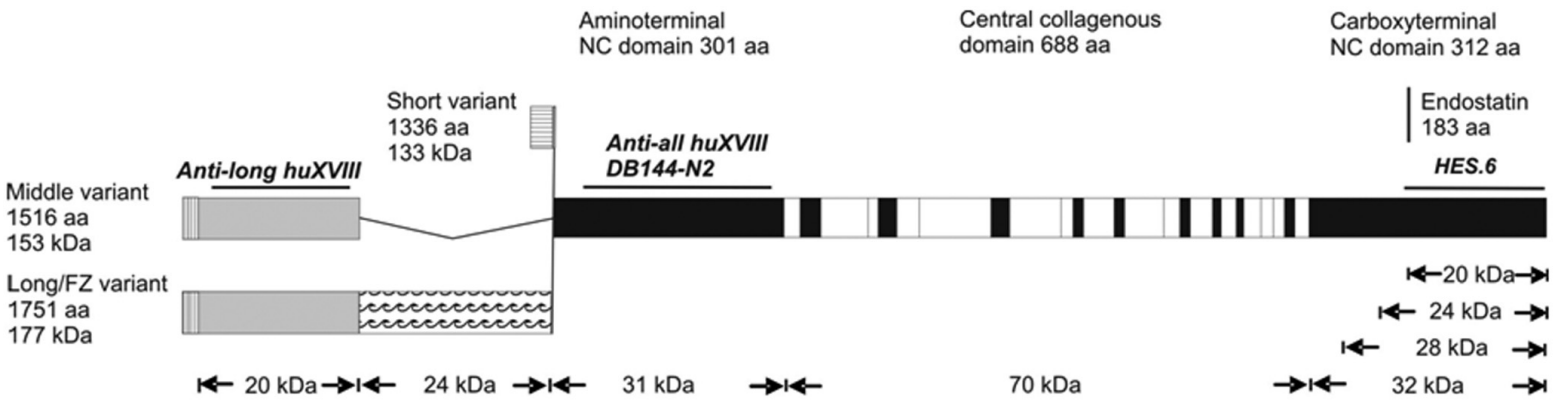
Schematic representation of the human collagen XVIII variants, termed as SHORT, MIDDLE and LONG/FZ. Collagenous sequences are shown in white. Non-collagenous (NC) amino terminal sequences common to all variants are shown in black. Non-collagenous amino terminal sequences common to the two long variants are shown in grey. A non-collagenous amino terminal sequence specific to the LONG/FZ variant is marked with waves. The amino acid lengths (aa) and the predicted molecular masses (kDa) of these sequences are given, as are those of the full length type XVIII collagen molecules. Signal sequences are indicated by vertical and horizontal hatching. The epitopes of the antibodies used in this work are shown by black horizontal lines. The molecular masses of some endostatin-containing carboxy-terminal fragments are also indicated.

There are at least 27 different species of collagen. Types I and III predominate within both healthy and fibrotic lung [6]. Perivascular tissue contains type XVIII collagen which is expressed as three variable polypeptide forms (SHORT, MIDDLE and LONG/frizzled) [7] (Figure 1). Endostatin is a 20 kDa proteolytic fragment of collagen XVIII. Recombinant endostatin has been shown to inhibit tumour growth and metastasis in animal models [8]. On the cellular level, endostatin specifically blocks growth factor-induced proliferation and migration of endothelial cells. The latter is proposed to involve integrin binding and subsequent disruption of the cell-matrix interaction either via Src tyrosine kinase/Rho pathway or mitogen activated protein kinase (MAPK)/p38 pathway [9-11]. Endostatin induces endothelial cell apoptosis in microgram doses [12], inhibits vascular endothelial growth factor (VEGF)-mediated signalling due to a direct interaction with VEGF receptor-2 [13], and inhibits cyclin D1 [14] and Wnt signalling [15]. The main focus of research into endostatin has been its anti-tumour effects. Recently, however, elevated levels have been found in the plasma of patients with preeclampsia, a condition also associated with pan-endothelial damage [16]. There is little information about the effects of endostatin on epithelial cells; however, it was shown to inhibit squamous cell carcinoma migration and invasion *in vitro *[17,18].

Resolution of ALI is dependent on the successful repair of a confluent barrier of alveolar epithelial and endothelial cells [19]. This process requires cell division and migration. Molecules that effect cell proliferation, migration and repair are therefore potential therapeutic agents in ALI [20]. As collagen degradation products are elevated in the lungs of patients with ALI [4], we hypothesised that endostatin may have a role in inhibiting lung repair in patients with ALI. The aim of this study, therefore, was to test the hypothesis that endostatin is elevated in the plasma and BAL of patients with ALI and to determine whether endostatin levels reflect the severity of injury and alveolar inflammation. In addition, we assessed changes in endostatin in OLV and LPS challenge models of early lung injury.

## Materials and methods

### Subjects

All patients enrolled in the study gave written informed consent themselves at the time of enrollment, or it was given by the consultant in charge of the intensive care unit (ICU) (not part of the research team) as their legal guardian if they were unable to give consent because of sedation and ventilation. In addition, in all cases, patients' next of kin gave informed signed assent to inclusion. In the UK. patients' relatives cannot consent for incapacitated relatives. In addition, those patients who recovered mental competency during their inpatient stay retrospectively provided signed consent for their inclusion in the study. This study was fully approved by the local National Health Service trusts and ethical review committee.

Patients were studied within 48 hours of admission to the ICU of Birmingham Heartlands and University Hospital, Birmingham, UK, between 2006 and 2008. 38 patients (22 male, mean age 62 years, standard deviation (SD) 16 years, 12 with direct and 21 with remote lung injury) were identified as having ALI according to the American-European consensus statement [21]. Patients were ventilated using pressure-controlled ventilation. Bronchoscopy and BAL was performed in all patients immediately following inclusion and, when possible, four days later (n = 22). The median time between patients satisfying the criteria for ALI and initial bronchoscopy was about six hours (range = 3 to 36) Of the patients who did not undergo repeat bronchoscopy, six had died, five had been extubated and five had contraindications. Ten ventilated patients with identifiable risk factors for ALI but who did not satisfy the consensus criteria for ALI were recruited as controls (6 male, mean age 59 years, SD 15 years). Ten non-smokers underwent bronchoscopy as normal controls.

Patient demographic characteristics were recorded at baseline. The acute physiology and chronic health evaluation II (APACHE II) and simplified acute physiology score II (SAPS II) were recorded as global markers of disease severity. The Murray lung injury score, partial pressure of arterial oxgen (PaO_2_): fraction of inspired oxygen (FiO_2_) ratio was recorded on the day of bronchoscopy. A summary of patient physiological severity is shown in Table 1.

**Table 1:** Characteristics of acute lung injury and septic at-risk patients

Characteristic	ALI	At risk for ALI	*P *value	95% confidence interval
PaO_2_:FiO_2 _ratio	130 (40)	255 (92)	0.001	75 to 167
Lung injury score	2.6 (0.4)	1.2 (0.3)	0.001	1.0 to 1.5
APACHE II	24.5 (8.2)	21.7 (8.9)	0.294	2.5 to 8.2
SAPS II	57.4 (17.5)	54.8 (12.8)	0.631	-8.3 to 13.5
SOFA score	8.9 (2.8)	6.6 (4.3)	0.116	-0.8 to 5.5

ALI = acute lung injury; APACHE = acute physiology and chronic health evaluation; FiO_2 _= fraction of inspired oxygen; PaO_2 _= partial pressure of arterial oxygen; SAPS = simplified acute physiology score; SOFA = sequential organ failure assessment. Data are expressed as mean (standard deviation).

#### Post-one lung ventilation patients

Ten patients (9 males, 1 female, age 63 years, mean forced expiratory volume (FEV) 1 94% predicted, mean 25 pack-year smoking history) undergoing elective oesophagectomy for oesophageal carcinoma underwent BAL of the collapsed lung at the end of the operation, which as such potentially suffers from ischaemia-reperfusion injury. Ventilation was performed with pressure control for eight patients and two patients with volume control. Mean peak airway pressures were 25.8 cm, standard error of the mean (SEM) 0.88 cm, and a mean tidal volume of 430 ml was achieved. Mean duration of OLV was 189 minutes, SEM 22.29 minutes. The preoperative PaO_2_:FiO_2 _ratio was a mean of 50.210, SEM 3.45, and immediate post-operative PaO_2_:FiO_2 _ratio was a mean of 40.2, SD 14.46, SEM 4.57, and average intraoperative FiO_2 _during OLV was 67%. None of these patients went on to develop early lung injury.

### Inhaled lipopolysaccharide-induced model of lung injury

We include data from a human LPS challenge study assessing the anti-inflammatory effects of simvastatin versus placebo in healthy volunteers. Data are presented from 10 patients treated with placebo (five males, mean age 26 years) who underwent bronchoscopy six hours post-inhalation of LPS. Full details of the patient characteristics and lavage fluid abnormalities are available in another paper [22].

### Bronchoscopy and bronchoalveolar lavage

Bronchoscopy was performed by instilling three 50 ml aliquots into the right middle lobe (median yield in ALI patients = 65 ml, IQR = 35 to 80 ml). Bronchoalveolar lavage fluid (BALF) was processed as described previously [23]. Into lithium heparin tubes (Becton Dickinson, London, UK), 15 ml of blood was collected simultaneously and stored on ice until processing.

### Lipopolysaccharide challenge

LPS (*Escherichia coli *serotype O26:B6; Sigma Chemicals, Poole, Dorset, UK) was dissolved in endotoxin-free sterile 0.9% saline and inhaled via an automatic inhalation–synchronised dosimeter nebuliser (Spira Electra 2, Hameenlinna, Finland), which delivers particles of a mass median aerodynamic diameter of 10 mm, as described previously. The dosimeter produces a calibrated aerosol of 8 μl at each slow inhalation starting from functional residual capacity to total lung capacity. Each subject performed five successive inhalations of the LPS solution (1.25 mg/ml) through the mouthpiece with a nose clip in place. The total dose of inhaled LPS was 50 μg. BAL was performed six hours after LPS inhalation according to standard guidelines by a single researcher (DM).

### Urea and protein determination

Urea concentration was determined using a commercially available urea kit (Sigma diagnostics, Poole, UK). The epithelial lining fluid (ELF) levels of endostatin were estimated using the following formula: ELF endostatin = BAL urea (mmol/L) × BAL endostatin/plasma urea concentration (mmol/L), as described previously [24]. BALF protein was measured using the Lowry method [25]. The protein permeability index was calculated as plasma protein/BAL protein [26].

### ELISA

Endostatin and IL-8 were measured by using an ELISA kit (R&D systems, Abingdon, UK) according to manufacturer's instructions. CV for intra-assay variability in BALF of endostatin is 3.6% and inter-assay variability was 8%. Spiking ALI BALF with recombinant endostatin standard (2.5 ng/ml) has a 96% recovery rate.

### Western blotting of BALF and plasma samples

Immunoprecipitation and western blotting of plasma and BALF was performed using methods described previously [27,28]. The specificities of the two polyclonal human collagen XVIII antibodies, anti-ALL huXVIII (QH48.18) and anti-LONG huXVIII (QH1415.7), have been verified earlier using competitive blot with the peptides that were used to generate these antibodies [7,27,29]. The following in house-developed antibodies were used in these studies in order to detect the precursor forms of type XVIII collagen and any degradation products: Anti-LONG huXVIII is a polyclonal anti-LONG human type XVIII collagen antibody which targets the N-terminal end of the two longer isoforms of XVIII collagen [30]. Anti-ALL huXVIII is a polyclonal human type XVIII antibody which targets the N-terminal end common to all three collagen XVIII isoforms [7]. A polyclonal anti-endostatin antibody known to detect proteolytically cleaved endostatin fragments [30].

### Western blotting of BALF samples

A 25 μl sample of BALF was loaded onto polyacrylamide gel and the proteins were separated on a 7 to 12% SDS-PAGE under reducing conditions, electrotransferred to a nitrocellulose membrane (Protran; Schleicher & Schuell, Dassel, Germany) and probed with rabbit polyclonal antibodies against endostatin (HES.6) [28] and human collagen XVIII (anti-all huXVIII QH4818 and anti-long huXVIII, QH1415.7) [7], all used at a concentration 1 μg/ml in 5% fat-free milk powder in 1 × PBS, followed by a horseradish peroxidase-conjugated goat anti-rabbit antibody (Bio-Rad, Abingdon, UK). After washing the membrane extensively with 1 × PBS/0.1% Tween, the proteins that were reactive to collagen XVIII and endostatin antibodies were visualised with ECL western blotting detection reagents (Amersham Pharmacia Biotech, Amersham, UK). For binding sites of antibodies to type XVIII collagen see Figure 1.

### Immunoprecipitation and western blotting of plasma samples

Collagen XVIII was immunoprecipitated from human plasma using a monoclonal anti-ALL type XVIII antibody, DB144-N2 bound to anti-mouse IgG-coated magnetic beads (Dynabeads M-280; Dynal, Malmo, Sweden), as described elsewhere [31]. The bound polypeptides were separated on a 7% SDS-PAGE under reducing conditions, electrotransferred to a nitrocellulose membrane and probed with rabbit polyclonal antibodies anti-all huXVIII (QH48.18) or anti-long huXVIII (QH1415.7) [7], both at a concentration of 1 μg/ml in 5% fat-free milk powder in 1 × PBS, followed by a horseradish peroxidase-conjugated goat anti-rabbit antibody. After washing the membrane extensively, the proteins reactive to collagen XVIII antibodies were visualised with ECL western blotting detection reagents. For binding sites of antibodies to type XVIII collagen see Figure 1.

### Statistical methods

The Ryan-Joiner normality test was used to test the distribution of the data. Non-parametric Mann-Whitney U tests were used for data that was not normally distributed (ELISA endostatin ALI plasma data and BALF data) and are quoted as medians (interquartile ranges (IQR)). Normally distributed data (optical density data for blots) are quoted as mean (SEM) and compared using analysis of variance (ANOVA). Statistical significance assumed at *P *< 0.05.

## Results

### Plasma levels of endostatin are elevated in patients with acute lung injury

**Figure 2:**
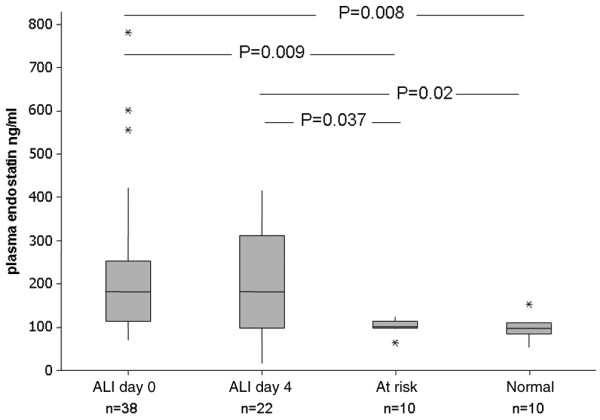
Plasma endostatin levels in patients groups. Endostatin was measured by ELISA. Endostatin is significantly elevated in patients with acute lung injury compared with normal and at-risk controls. ALI = acute lung injury.

Median plasma levels of endostatin were elevated in patients at the onset of ALI (182 ng/ml, IQR = 111 to 244) compared with normal controls (median 96.3 ng/ml, *P *= 0.008) and patients at risk from ALI (102 ng/ml, *P *= 0.0094). On day four, plasma levels in ALI patients remained elevated (182.8 ng/ml, IQR = 97.8 to 284.6; Figure 2).

There were significant linear associations between plasma endostatin at the onset of ALI and the global severity of illness markers APACHE II (r = 0.37, *P *= 0.038) and SAPS II (r = 0.44, *P *= 0.007) but not with lung injury score (r = -0.3, *P *= 0.9) (data not shown). Plasma endostatin levels at the onset of ALI or at day four did not predict whether patients subsequently survived or died.

### BALF endostatin is significantly elevated compared with normal and at-risk patients

**Figure 3:**
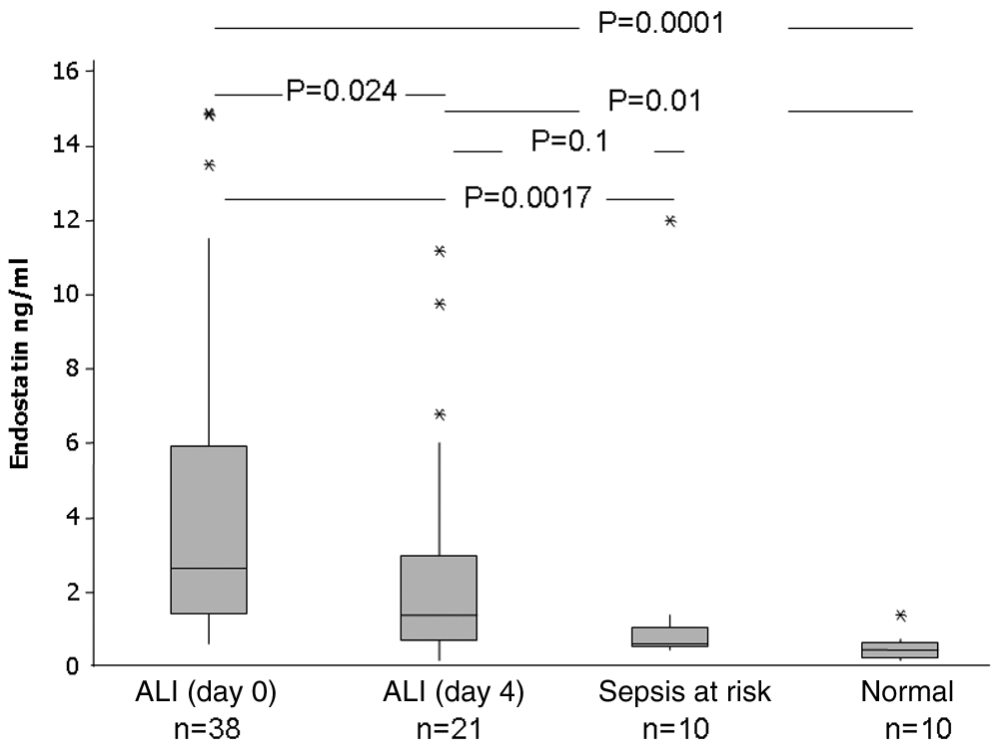
BALF endostatin in different patient groups. Bronchoalveolar lavage fluid (BALF) endostatin is significantly elevated in the BALF of patients with acute lung injury compared with normal and at-risk controls. Lavage was repeated where possible again at day 4 in 23 patients. Levels fell significantly from day 0 to day 4 but remained elevated compared with normal and at-risk controls. ALI = acute lung injury.

ALI patients had greater endostatin levels in BALF on both day 0 (median ALI 2.6 ng/ml, IQR = 0.24 to 0.64, *P *= 0.001) and day 4 (median ALI 1.36 ng/ml, IQR = 0.72 to 2.63, *P *= 0.01) than healthy individuals (0.08 ng/ml). ALI day 0 endostatin levels (2.6 ng/ml) were higher than those at risk of ALI (0.6 ng/ml, IQR = 0.3 to 1.4, *P *= 0.0017; Figure 3).

Endostatin levels in ALI BALF fell from day 0 to day 4 (day 0 median 2.6 ng/ml, IQR = 1.4 to 5.1; vs day 4 median 1.36 ng/ml, IQR = 0.76 to 2.9; *P *= 0.02). There was no difference between BALF levels at day 0 or day 4 between patients who died or survived, or those with direct or remote lung injury. BALF endostatin did not correlate with APACHE II, SAPS II or lung injury score at day 0 or day 4.

### ALI BALF endostatin correlates with BAL neutrophilia and protein permeability index

There was also a significant relationship between day 0 BALF endostatin levels and protein permeability index (r = 0.441, *P *= 0.013). In addition, day 0 BALF endostatin levels correlated with the degree of BAL neutrophilia (r = 0.417, *P *= 0.034) but not with total cell count or BALF IL-8. At day 4 endostatin also correlated strongly with protein permeability index (r = 0.723, *P *= 0.002) and total neutrophil counts (r = 0.76, *P *= 0.007) (data not shown).

### Plasma endostatin is higher than ELF endostatin

Plasma levels of endostatin were significantly higher than calculated ELF levels in the lung (median 182 ng/ml vs ELF 119 ng/ml, 95% confidence interval (CI) diff = 18 to 169; *P *= 0.016). When ELF levels were lower than plasma levels there was a significant correlation (r = 0.47, *P *= 0.001) between plasma and ELF endostatin at the onset of ALI (data not shown). However, in some patients, calculated ELF levels were actually higher than plasma suggesting intra-alveolar generation of endostatin. Interestingly, at day 4 ELF endostatin correlated with lung injury score (r = 0.499, *P *= 0.049) but not in ALI patients at day 0.

### OLV and LPS challenge induce a BAL neutrophilia and increase PPI

OLV and LPS challenge induced a BAL neutrophilia and an increase in protein permeability index (PPI) compared with normal controls (PPI in OLV: median = 0.00636, IQR = 0.00389 to 0.01188; *P *= 0.013 vs normal; PPI in LPS: median = 0.0025, IQR = 0.0013 to 0.003, *P *= 0.011 versus normal; normal PPI = 0.0014, IQR = 0.0008 to 0.0024). Percentage of neutrophils in BAL rose from 1.1% (IQR = 0.0 to 2.65) in normal BALF to median 11% (IQR = 2 to 60.5) in OLV (*P *= 0.011), and 41% (IQR = 24 to 57) in LPS challenge as described previously [22].

### OLV and LPS challenge increases BALF endostatin

**Figure 4:**
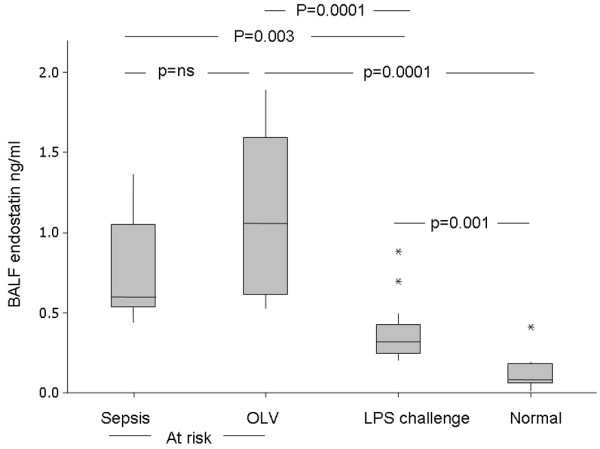
BALF endostatin in the different at-risk groups compared with normal individuals. BALF = bronchoalveolar lavage fluid; LPS = lipopolysaccharide; OLV = one lung ventilation.

Inhalation challenge with LPS increased BALF endostatin from a median of 0.08 ng/ml in normal individuals to 0.385 ng/ml (IQR = 0.285 to 0.448, *P *= 0.001) in LPS-challenged patients. OLV increased BAL endostatin (median compared with normal and LPs challenge; Figure 4). BALF endostatin correlated with PPI in the LPS challenge (r = 0.63, *P *= 0.006) but not in OLV patients. In contrast, plasma levels of endostatin were not altered by LPS challenge or OLV (data not shown).

### Type XVIII collagen precursors are elevated in both the plasma and BALF of patients with ALI compared with normal controls

**Figure 5:**
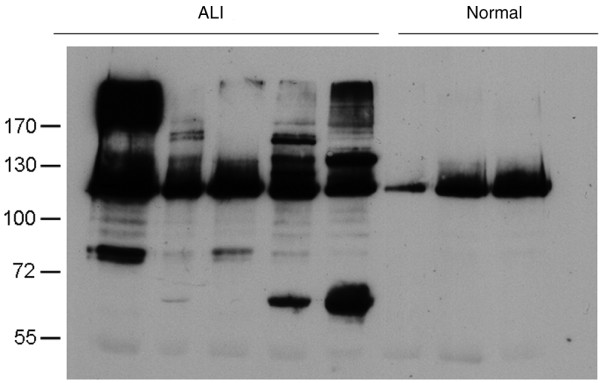
Immunoprecipitation with anti-ALL antibody and detection with anti-LONG huXVIII antibody. Elevated type XVIII collagen precursors in the plasma of ALI patients compared with normal controls is shown. ALI = acute lung injury.

In exploratory experiments performed in five consecutive non-selected patients, plasma and BALF from patients with ALI demonstrated increased density and number of fragments of the circulating precursor forms of type XVIII collagen compared with normal controls. Whole lane densitometric analysis of the western blot by anti-LONG antibody confirmed increased plasma precursors in ALI (mean optical density (OD) = 198, SE 38) compared with normal plasma (mean OD 79.8, SE 7.9, 95% CI = 11.6 to 226, *P *= 0.037) (representative gels are shown in Figure 5). Western blotting of the precipitated proteins with three different antibodies to human collagen XVIII (anti-ALL, anti-LONG and anti-endostatin) revealed a main 120 kDa band, which is presumably derived from the middle variant of collagen, containing the N-terminus and the collagenous central part of the molecule but lacking the endostatin domain (Figure 5 and data not shown). Based on the current knowledge of the structure and properties of collagen XVIII, the diffuse bands over 170 kDa in the plasma from some ALI patients are interpreted to represent the long, potentially glycosylated, forms of collagen XVIII.

**Figure 6:**
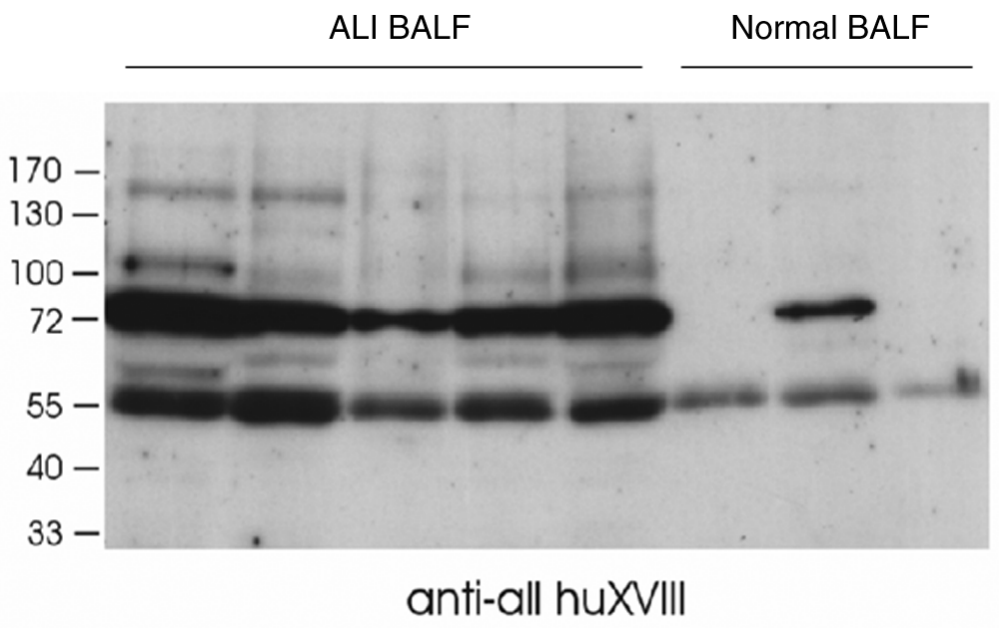
Western blot by anti-ALL huXVIII antibody. It shows elevated type XVIII collagen precursors in the bronchoalveolar lavage fluid (BALF) of patients with acute lung injury compared with normal controls. ALI = acute lung injury.

There was also a clear increase in collagen XVIII fragments in BALF detected by the anti-ALL antibody revealing a mean adjusted whole lane OD of 94.9 (SE 9.3) in ALI compared with 41.21 (SE 4.8) (95% CI = 26.6 to 80.6; *P *= 0.004) in normal controls (Figure 6). The anti-ALL antibody does not bind the carboxy end of type XVIII collagen, so this suggests that type XVIII collagen precursors are present within the BALF of patients with ALI. Similarly, using the anti-LONG antibody, there was evidence of increased BALF levels of the long forms of collagen XVIII (ALI OD = 54.74 (SE 2.1), normal OD = 40.39, 95% CI = 6.8 to 21.7, *P *= 0.004) (data not shown). The main bands of 55 kDa, also detectable with anti-LONG antibody, and 72 kDa were interpreted to represent proteolytic N-terminal fragments from the middle form and the short form of collagen XVIII, respectively (Figure 5 and data not shown).

### BALF from ALI patients contain multiple endostatin-like fragments

Western blotting of BALF with the HES.6 antibody that recognises the carboxyl (endostatin) end of type XVIII collagen revealed increased levels of C-terminal endostatin-containing fragments of collagen XVIII are present within the BALF of ALI patients with a fragmentation pattern that was different to normal controls.

**Figure 7:**
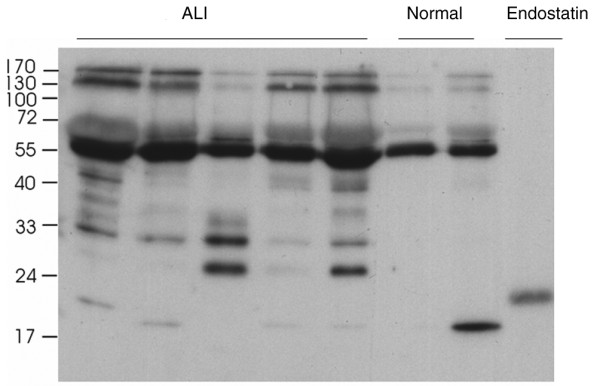
Western blot by anti-endostatin antibody HES.6. It shows endostatin-like degradation products in bronchoalveolar lavage fluid (BALF) of patients with acute lung injury. Positive control was a recombinant human endostatin carrying a histidine tag (molecular weight of 22 kda). ALI = acute lung injury.

Control samples contained a strong 55 kDa band extending to the central collagenous domain, full-length collagen XVIII precursors between 130 and 170 kDa, and some samples also showed a clear 20 kDa endostatin band. In addition to these bands, ALI samples showed a pattern of endostatin-containing fragments varying in size from 22 to 50 kDa. Histidine-labelled recombinant endostatin was used as a positive control (Mw 22 kDa). Whole lane densitometric analysis showed that significantly elevated amounts of type XVII carboxy terminal endostatin-like fragments were present in ALI samples (mean OD = 54, SE 3) compared with normal BALF (mean OD = 43.9, SE 3.87, 95% CI = 2 to 19, *P *= 0.02) (representative gel shown in Figure 7).

## Discussion

This study has demonstrated that endostatin is elevated within the plasma and BALF of patients with ALI. Endostatin levels in ALI BALF reflected the degree of neutrophilia and the extent of the loss of protein selectivity of the alveolar-capillary barrier. Plasma levels of endostatin at the onset of ALI were associated with the severity of physiological derangement. Western blotting confirmed elevated type XVIII collagen precursor levels and multiple amino- and carboxy-terminal fragments in plasma and BALF. Increases in endostatin occur early after OLV and LPS challenge in human volunteers but this is compartmentalised to the lung.

Endostatin was detectable in the plasma of all normal subjects studied suggesting a physiological role in the homeostasis of angiogenesis. Elevated endostatin levels have previously been reported in the plasma of patients with preeclampsia, a condition associated with pan-endothelial damage [16]. Similarly, in this study, median plasma level of endostatin in ALI patients were elevated in comparison to both those at risk and normal controls. Plasma levels of endostatin correlated with the severity of the systemic physiological insult rather than lung injury *per se*. The observed relationship between BALF endostatin and protein permeability index, and the apparent plasma endostatin: ELF endostatin gradient in most of our patients further suggest that alveolar levels of endostatin are raised at least in part because of leakage from the microvasculature into the alveolus at sites of inflammation of the alveolar-capillary membrane. However, in some patients, calculated ELF levels were higher than plasma supporting a role for alveolar generation of endostatin from the type XVIII collagen precursors that we have shown are present.

Endostatin was also elevated in the BALF but not plasma of patients with sepsis who were at risk of ALI. In order to study the time course of changes in endostatin, we evaluated changes in response to two injurious stimuli: OLV and LPS challenge. Both these stimuli induce BAL neutrophilia and increased the PPI in the volunteers/patients. Endostatin was elevated in the BALF but not plasma of the patients after an average of 2.5 hours of OLV and after six hours in the LPS-challenged patients. Thus, endostatin release appears to be compartmentalised to the lung early after such localised injurious stimuli. The relationships of BALF endostatin with PPI in these *in vivo *models of the early response to ALI and in established ALI suggest that endostatin generation might be useful as a measure of alveolar capillary damage and support a potential pathophysiological role.

Endostatin is derived from collagen XVIII which is a major proteoglycan of both endothelial and epithelial basement membrane zones. Specialised capillaries, such as lung alveoli, express type XVIII collagen in their basement membranes. Three forms of collagen XVIII precursor, designated the SHORT, the MIDDLE and the LONG/frizzled variants, are differentially expressed in normal human tissues. The SHORT form predominates in vascular basement membranes [7], whereas the MIDDLE and the LONG forms are abundantly expressed in hepatocytes and are circulating plasma proteins [31,32]. Furthermore, proteolytic degradation of type XVIII collagen can generate multiple carboxy-terminal fragments of precursor collagen XVIII [33,34]. Clearly, therefore, elevated endostatin levels could arise because of cleavage of elevated circulating LONG forms and/or cleavage of the SHORT form at sites of inflammation within the alveolar-capillary membrane.

Our immunoprecipitation and western blot results suggest that degradation products of the LONG type XVIII collagen are elevated in both the plasma and BALF of patients with ALI. Proteolytic cleavage of the carboxy end of collagen XVIII has been demonstrated for many enzymes including elastases, cathepsins and matrix metalloproteinases (MMP), which have been implicated in the pathogenesis of ALI [28,34,35]. Western blotting of ALI BALF with antibody against the carboxy-terminal of collagen XVIII demonstrated multiple endostatin-like fragments similar in size to those produced by MMP degradation. These fragments may be important because they are known to be bioactive and inhibit β-fibroblast growth factor-induced endothelial cell proliferation and migration [28].

What are the implications of our findings for alveolar capillary repair in ALI? Previous studies using the animal corneal micropocket assay have demonstrated that BALF has a strong angiogenic potential that is related to elevated CXC chemokine levels [36]. In contrast, when looking at human primary lung microvascular endothelial cells, ALI BALF caused cell death in a TNF- and oncostatin-dependent manner [37]. Given the known anti-endothelial actions of endostatin, the elevated levels of endostatin seen in our patients may play a role in such endothelial toxicity.

In addition to effects on endothelial cells, we have recently reported that endostatin inhibits the proliferation and *in vitro *wound repair responses of both distal small airway epithelial cells, and primary human type II epithelial cells [38]. Thus elevated levels of endostatin within the lung may also play a role in aberrant epithelial repair mechanisms in ALI. The observed relationships in this study between endostatin levels, the degree of neutrophilic inflammation and physiological severity suggest the multiple cellular effects of endostatin within the lung might be of clinical importance.

This study has several limitations. Firstly, despite several attempts, we were unable to detect plasma endostatin fragments by immunoprecipitation and western blotting with the HES.6 antibody, which appears unsuitable for plasma endostatin estimation.

Secondly, there were a number of drop outs in our sequential assessments, for clinical reasons (extubation, death or contraindication to bronchoscopy). We do not believe the results have been biased by this because there were no baseline differences in endostatin between those re-studied at day 4 and those not. Thirdly, it would be interesting to observe the effect of neutralising endostatin bioactivity in BALF on endothelial cell viability. Unfortunately, no effective specific inhibitor for the bioactivity of endostatin is currently available as endostatin has a wide variety of effects on endothelial cells influencing nearly 12% of the genome via a variety of mechanisms [39]. Clearly in order to firmly establish the pathophysiological importance of endostatin release in the development of lung injury further study in animal knock-out models would be required.

## Conclusions

To our knowledge, this is the first report of the presence of endostatin in plasma and BALF of humans with ALI. It is clear that ALI patients have persistently elevated alveolar endostatin levels during the early course of the disease. Our models of the early onset of lung injury suggest these changes occur very early in the injurious process. As endostatin may adversely affect both alveolar-barrier endothelial and epithelial cells, its presence within both the circulation and the lung may have a pathophysiological role in ALI that warrants further evaluation.

## Key messages

• Endostatin is elevated within the plasma and BALF of patients with ALI.

• Endostatin levels in ALI BALF reflected the degree of neutrophilia and the extent of the loss of protein selectivity of the alveolar-capillary barrier.

• Plasma levels of endostatin at the onset of ALI were associated with the severity of physiological derangement.

• Western blotting confirmed elevated type XVIII collagen precursor levels and multiple amino- and carboxy-terminal fragments in plasma and BALF.

• Increases in endostatin occur early after OLV and LPS challenge in human volunteers but this is compartmentalised to the lung.

## Abbreviations

ANOVA: analysis of variance; ALI: acute lung injury; APACHE II: acute physiology and chronic health evaluation II; BAL: bronchoalveolar lavage; BALF: bronchoalveolar lavage fluid; ELF: epithelial lining fluid; ELISA: enzyme-linked immunosorbent assay; FEV: forced expiratory volume; FiO_2_: fraction of inspired oxygen; ICU: intensive care unit; Ig: immunoglobulin; IL: interleukin; IQR: interquartile range; LPS: lipopolysaccharide; MAPK: mitogen activated protein kinase; MMP: matrix metalloproteinases; OD: optical density; OLV: one lung ventilation; PaO_2_: partial pressureof arterial oxygen; PBS: phosphate-buffered saline; SAPS II: simplified acute physiology score II; SD: standard deviation; SEM: standard error of the mean; TNF: tumour necrosis factor; VEGF: vascular endothelial growth factor.

## Competing interests

The authors declare that they have no competing interests.

## Authors' contributions

GDP, NN, SS, DM, AR, WT, MM and DRT recruited patients and performed bronchoscopy. GDP, NN and AR performed ELISA measurements. RH performed the western blot analyses. All authors contributed to writing the paper. GDP NN and DRT performed the statistical analyses. NN and GDP contributed equally to the paper.
